# Efficiency in Kinesiology: Innovative Approaches in Enhancing Motor Skills for Athletic Performance

**DOI:** 10.3390/jfmk8030111

**Published:** 2023-08-04

**Authors:** Vincenzo Sorgente, Diego Minciacchi

**Affiliations:** Kinesiology and Motor Control (Ki.Mo.Co.) Laboratory, Department of Experimental and Clinical Medicine, Physiological Sciences Section, University of Florence, 50134 Florence, Italy; diego.minciacchi@unifi.it

The inaugural edition of the Special Issue titled “Efficiency in Kinesiology: Innovative approaches in enhancing motor skills for Athletic Performance” has been effectively concluded.

In recent years, sport science research has substantiated the efficacy of certain modalities as the paramount outline for assessing, enhancing, and even prognosticating athletic performance [1,2]. These methodologies encompassed both bio-motor (e.g., power development) and related technical components (e.g., vertical jump during a basketball shoot). However, the scientific and technological progress keeps giving rise to further potential opportunities for optimizing the core principles which craft the best sporting performances. In order to corroborate this enhancement, various approaches have been employed, spanning from the ecological, lab-independent, cost-effective, to the minimal invasiveness ones. The perspective of upgrading well-known and validated applications is indeed intriguing to the scholarly community as well as the in-field practitioners (i.e., trainers and athletes), both dedicated to the unceasing improvement of athletic conditioning [3]. Thus, the conceptual groundwork of the present volume is conceived on the need to address these novel strategies and proposals through a methodical and cohesive fashion. This Special Issue welcomes 13 original research articles plus one case report, centered on implementing cutting-edge approaches to efficiently sharpen motor skills in the function of elevating sporting performances. The athletic domains covered by this Special Issue include soccer (situational performances for both male and female competitors), swimming (exercise physiology), wrestling (sociocultural aspects), basketball (new test validation), volleyball (new test validation and exercise kinematics), handball (exercise kinematics), fencing (visual strategies), other than non-sport specific biomechanics, strength and conditioning, and robotics.

Present soccer demands are increasing in terms of running requirements and the augmenting number of scheduled matches provide several periods of fixture congestion during the season. Strategic and hyper-specific use of the team’s resources is becoming a must for competitive success. With the use of GPS technology, Muñoz-Castellanos et al. [4] monitor accumulated workloads during the season, seeking for differences among roles played, and between starters versus substitutes. They find that each position (central defender, full back, midfielder, wide midfielder, striker) shows specific behaviors in distance covered during a congested competitive period. The Authors conclude that coaches should pay attention to the fatigue produced by the number of high decelerations and that an individualized training protocol should be considered according to the running requirements of each position on the pitch. Additionally, Furtado Mesa et al. [5] show that seasonal accumulated total distance, sprints, and high-speed distance are significantly greater for starters than substitutes. Also, accumulated training load and training load per minute played in matches do not differ between starters and substitutes as the accumulated training load profiles of substitutes is similar to that of starters.

Evaluating force–velocity characteristics on dry land is of the utmost importance in swimming, since higher levels of these bio-motor abilities positively affect in-water performance. Sorgente et al. [6] search for differences among swimmers’ stroke (butterfly, backstroke, breaststroke, front-crawl) and distance specialization (50, 100, and 200 m) by measuring the maximum force–velocity exertion during the pull-up motion. Assessments are performed (via linear encoder) before and after taking part in an official swimming race. Both force and velocity are significant predictors of swimming race time. Sprinters (50 m and 100 m) of all strokes exert significantly higher force–velocity compared with 200 m swimmers. Interestingly, breaststroke sprinters present significantly lower force–velocity compared to sprinters specialized in the other strokes. Searching for the role of stroke and distance specializations in modeling swimmers’ force–velocity abilities can heavily influence swimming training and performance.

Employing the Parental Support Scale for Children in Sports, Biletic et al. [7] investigate the effects of age and popularity of wrestling in influencing perceived parental support. During the period of entry into specialization, children perceive less parental support and lower parental belief in the benefits of sport practicing. Moreover, as could be expected, in environments in which wrestling is popular, parents know the sport better and can actively participate; therefore, children perceive more parental support. These outcomes may help coaches to better understand the athlete–parent relationships and correlated psychological aspects during sensible stages of the young athlete’s development.

Jumping ability in basketball is assessed using standardized vertical jump tests which, however, lack specificity by not considering the player’s basketball skills. Theodorou et al. [8] propose the pivot step jump test (PSJT) as a novel test designed to evaluate the jumping abilities of basketball players by combining a pivot step on one leg with a maximum bilateral vertical jump. To this scope, intra-and intersession reliability and validity are evaluated (performing the PSJT and a series of criterion jumping tests). No changes are found in PSJT performance between test sessions and excellent intra- and intersession reliability was observed. Furthermore, correlation coefficients indicate high factorial validity between the jumping tests and PSJT. Therefore, PSJT offers a valid assessment of jumping ability in basketball, having the practical potential to assess sport-specific jumping skills in young basketball players.

Another innovative test is proposed in this Special Issue for the sport of volleyball. Đolo et al. [9] aim to determine the test–retest reliability and discriminative ability of five sport-specific kinesthetic differentiation tests in volleyball female players. In particular, kinesthetic differentiation ability is determined by evaluating (1) overhead passing, (2) forearm passing, (3) float service with a net, (4) float service without a net, (5) float service 6 m from the net. Parameters of the intraclass correlation coefficient are excellent in all tests except for the float service with the net, whose reliability was good. Hence, the Authors endorse this specific battery test as a reliable tool to monitor kinesthetic differentiation ability in female volleyball players.

The second volleyball-centered paper of this Special Issue focuses on the relationships among ankle flexibility, knee extensors torque, and performance in countermovement jump (CMJ) by Panoutsakopoulos and Bassa [10]. Testing includes the CMJ with–without an arm swing, and—on an isokinetic dynamometer—maximal knee extensions and flexions at three angular velocities. CMJ height and relative power are positively correlated with the extensors’ torque at 180°/s and are negatively correlated with the flexibility level of the dominant ankle, also revealing that more flexible players jump significantly higher during the CMJs. The Authors conclude that a more flexible ankle joint and a higher isokinetic knee extensor’s torque generating capacity result in higher CMJ performance. Therefore, training of ankle flexibility should be emphasized, and specific screening should be included during preseason in youth female volleyball players.

Sport-specific kinematics is further explored in handball. In particular, Burger et al. [11] study the kinematic parameters of single side feint movement between elite and professional level handball players. In handball, the feint movement is a fundamental technical and strategical element for offensive players to outplay their guard and score. The kinematic analysis is conducted using a GAIT—LaBACS software system (ver. 1.0), considering seven kinematic parameters for the “feint” and “actual” phases recorded by BASLER-402-FC and PANASONIC VW-D5100 cameras. Two variables have significant differences between elite and professional players: (1) step length of the stride leg; (2) moving the leg opposite to the throwing arm, demonstrating that less skilled players use more space for the same technical element.

Advancements in technology enable quantification of wide-ranging features of human movement. At variance from the study by Theodorou et al. [7] which quantifies sport-specific abilities of basketball players, here Philipp et al. [12] investigate technologies’ reliability prior to comparison with established industry gold standards. The Authors seek to determine the inter-device reliability between two identical markerless motion capture systems placed in close proximity (3D-MCS, DARI Motion; each composed by eight high-definition cameras recording at 60 fps). The test comprises 29 different elementary movement tasks. The results indicate negligible or small between-device effect sizes, while showing mostly excellent, moderate, or better agreement when looking at the ICC values, and little differences as for metrics measuring joint angles and distance measures. The preliminary though promising nature of the data leads the Authors to suggest that 3D-MCS may provide practitioners with a new opportunity to measure the movement characteristics of athletes reliably and efficiently.

Innovative approaches for assessing and ameliorating sport-specific performance are inquired in fencing. Bagot et al. [13] investigate the visual activity of fencers in conditions resembling official competitions. Eight national level fencers are recruited. Measures are performed by a head-mounted Pupil Invisible Eye tracking device (Pupil Labs^®^, Berlin, Germany) during the simulated bouts. Findings indicate that the main fixation in foil and sabre is the upper torso, while in epee, it is the lower torso. Two additional areas of interest are identified: (1) the score machine; (2) an area involving fixations that do not target a specific area of their opponent. Although these two areas are not directed towards the opponent, they still testify to a visual activity performed during a competition and potentially reflect what happens in a real match. Conversely, the study finds no direct link between visual activity and performance. The Authors conclude that fencers adapt their visual search strategy to the fencing specialization, i.e., the ruleset, that they take part in.

Previous research investigating the association of strength performance and anthropometric variables is often performed in a sample of pooled sexes or one sex only or by utilizing tests with low ecological validity. As such, Falch et al. [14] conducted a randomized cross-over study investigating the association of anthropometrics with strength performances in the squat and bench press for resistance-trained adult males and females and whether the association differed between the sexes. Participants are tested for strength performances with 60% of their 1-RM in the squat and bench press. The Authors find that some associations between strength performance and anthropometric variables differ between males and females. Namely, in the AMRAP (as many reps as possible) squat, thigh length is inversely associated with performance in males, while fat percentage is inversely associated with performance in females. However, for both sexes, lean mass and body height are associated with 1-RM strength in the squat and bench press, while body height is inversely associated with AMRAP performance.

Apart from squat jumps, CMJ, and drop jumps, differences among other jump variations are not sufficiently known, making it difficult to select data-driven exercises. To address this gap, Janikov et al. [15] compare specific concentric and eccentric jump parameters of maximal effort CMJ, jumps over 50 cm hurdle (HJ), and jumps onto a 50 cm box (BJ). The data (average of the three repetitions of each jump, performed on separate days) are collected using force platforms and a linear position transducer. No differences are found in peak velocity, peak vertical and resultant force, and total impulsion time. The Authors stress how overall training load could decrease dramatically when performing BJ, because of the half-reduced peak impact force compared to CMJ and HJ.

Schönau et al. [16] investigate whether the amplitude-force relationship of back muscles could be altered systematically by using different training modalities. Graded submaximal forces on the back are applied by defined forward tilts in a full-body training device (CTT Centaur, BfMC). Surface EMG is recorded utilizing a monopolar 4 × 4 quadratic electrode scheme in the lower back area. Between-group tests reveal significant differences between strength trained subjects vs. both endurance-trained and physically inactive subjects, at both the medial and caudal electrode positions. These results point towards training-related changes to the fiber-type composition of muscles in the strength-trained participants, particularly for their paravertebral region.

The Special Issue closes with an insight into the innovative use of robotics for neuromotor rehabilitation in clinical settings. Koseki et al. [17] provide an ankle rehabilitation training program with robot-assisted device in a patient with incomplete spinal cord injury. Using a three-dimensional motion analyzer and surface EMG, the Authors evaluate the treatment effectiveness using ankle plantar dorsiflexion exercises in the sitting position, knee flexion—extension exercises in the standing position, and stepping exercises in the standing position with hybrid-assistive limb assistance. Before rehabilitation, the patient was unable to perform voluntary ankle movements due to severe motor–sensory dysfunction. The training program is able to induce muscle potentials in the left tibialis anterior muscle during plantar dorsiflexion of the ankle.

Finally, we wish to gratefully acknowledge the essential contributions from all the Authors, Reviewers, and Editors towards this Special Issue.

Given the great success of this Special Issue, we have launched a second edition. We believe that this subject holds the potential to drive advancements in sports science by bridging cutting-edge scientific research with the on-field training methodologies and experiences.
